# Novel spectrofluorimetric approach for determination of ledipasvir through UV-irradiation: application to biological fluids, pharmacokinetic study and content uniformity test

**DOI:** 10.1039/c9ra07949a

**Published:** 2019-10-24

**Authors:** Mohamed A. Abdel-Lateef, Ramadan Ali, Mahmoud A. Omar, Sayed M. Derayea

**Affiliations:** Department of Pharmaceutical Analytical Chemistry, Faculty of Pharmacy, Al-Azhar University Assiut Branch Assiut 71524 Egypt m_aldoyik@yahoo.com mohamedgad-alkarim@azhar.edu.eg; Department of Analytical Chemistry, Faculty of Pharmacy, Minia University Minia 61519 Egypt; Department of Pharmacognosy and Pharmaceutical Chemistry, College of Pharmacy, Taibah University Medinah Saudi Arabia

## Abstract

A highly sensitive and specific fluorescence dependent approach was created for quantitation of a recently approved anti-HCV drug (ledipasvir). This approach relies on the innovative enhancement in fluorescence intensity of ledipasvir upon exposing the cited drug to direct UV irradiation as a photo-physical fluorescence enhancer. The fluorescence of the resultant solution was measured at an emission peak of 375 nm (321 nm excitation). The photoluminescence properties of the resultant product were carefully examined and the quantitative rectilinear concentration plot for the method was 5.0–150.0 ng mL^−1^ with a detection limit of 0.9 ng mL^−1^ and a quantitation limit of 2.7 ng mL^−1^. The excellent analytical features of the proposed method allow to the specific and sensitive estimation of ledipasvir either in plasma samples or in tablet dosage form without any interference from pharmaceutical excipients or other co-formulated anti-HCV drugs (sofosbuvir). The developed analytical and bio-analytical procedures were created and validated according to ICH guidelines and FDA guidelines, respectively. Since the main elimination route for ledipasvir is *via* faecal excretion, the studied drug was determined for the first time in faecal samples by the method with adequate recovery. Moreover, the pharmacokinetic parameters (*C*_max_, *t*_max_, *t*_1/2_, AUC_0–*t*_, and AUC_0–∞_) for ledipasvir were determined by the proposed method. Additionally, the proposed method was successfully applied for supervising the content uniformity for ledipasvir in its pharmaceutical tablets.

## Abstract 2

Enhancement in the fluorescence spectra of ledipasvir after exposing to strong UV irradiation.

## Graphical abstract

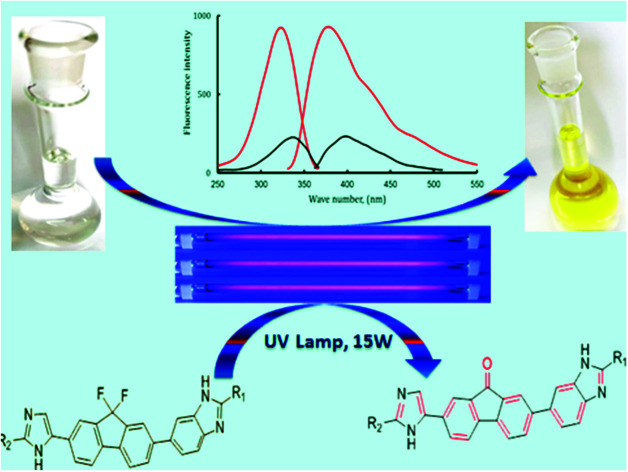

## 1. Introduction

**Fig. 1:**
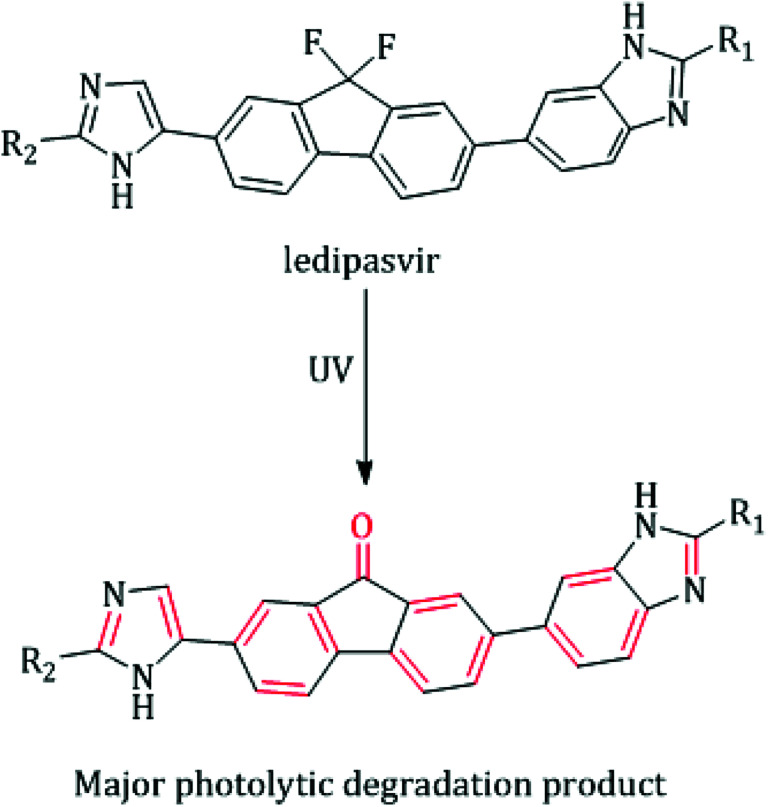
Suggested UV fluorescence enhancement mechanism for LDS.^9,13^

Approximately three percent of the world's population suffer from infection with chronic hepatitis C virus (HCV). Globally, at least one-third of hepatocellular carcinoma cases are attributed to chronic HCV-infection, and about 350 000 people die from complications caused by HCV-related diseases (ascites, cirrhotic liver, and liver dysfunction) per year.^1,2^ HCV can be classified into six genotypes, and more than fifty subtypes.^3^ About sixty percent of the infected people endure the strain of HCV genotype 1.^3,4^ It is predominant in Japan, North America and Eastern, Southern and Northern Europe.^3^ Ledipasvir (LDS, Fig. 1) is a recently FDA approved NS5A inhibitor with effective antiviral strength against all strains of HCV genotypes 1.^5^ Due to its recent introduction in the market, few techniques were reported for LDS estimation including chromatographic,^6–9^ spectrophotometric^10,11^ and spectrofluorimetric methods.^12–14^ Although the reported chromatographic techniques are sensitive enough,^6–8^ they are subject to extraction procedures as the plasma samples contain a high content of phospholipids as well as it requires expensive organic solvents and long operating time.^15^ In addition, all the reported UV methods were restricted to the estimation of LDS in the tablets dosage only without extension to biological fluids analysis due to their limited selectivity and sensitivity.^10,11^ The spectrofluorometric technique is one of the well-known analytical tools that provide the goal of increasing the method simplicity, selectivity and sensitivity without loss of precision.^16^ In spite of the reported spectrofluorimetric methods^12–14^ were applied for the determination of LDS in the plasma and tablets but they not extended to pharmacokinetic study and content uniformity test of LDS by this technique. Moreover, up till now the determination of LDS in the faecal samples (the major excretion route for LDS^5^) was not reported by any technique. Hence, the authors' attention was to develop a novel luminescent approach with higher specificity and sensitivity for the analysis of LDS either in biological fluids or in the tablets dosage form. Until now, no issued article was concerned with applying the UV radiation as a photo-physical analytical tool to enhance the spectrofluorimetric analysis of medicinal drugs. The proposed fluorimetric method utilizes an attractive idea, which relies on exposing LDS solution to strong UV radiation. Consequently, LDS will be converted to highly fluorescence product (fluorenone derivative), which allow to a direct, specific and highly sensitive estimation of LDS without interferences from other drugs (*e.g.* sofosbuvir (SOF)), excipients or biological matrices.

## 2. Experimental

### 2.1. Apparatuses

UV Trans-Illuminator, a model of WUV-L50 (dual wavelengths of 365 and 312 nm) containing six UV lamps with lamp power of 15 watts (Witeg Labortechnik GmbH, Am Bildacker, and Wertheim, Germany) was used for UV irradiation purposes (the device was adjusted on the button of all wavelengths). Fluorescence spectrometer FS2 (Scinco, Korea), was utilized for all spectrofluorimetric measurements (the device was adjusted at 570 nm min^−1^ for the scanning rate). VM 300 Supermixer vortex, Precisa XB 220 digital balance, Falcon™ (15 & 50 mL) Conical Tubes, and borosilicate glass volumetric flasks (10 mL) were utilized throughout this work. Also, L-500 tabletop low-speed centrifuge (Changsha Xiangyi Centrifuge Instrument Co., Changsha, China) and Pico 21 centrifuge (Thermo Electron LED GmbH, Osterode, Germany) were utilized for centrifugation purposes.

### 2.2. Chemicals and materials

LDS and SOF (purity of 99.5% and 99.4% w/w respectively) powders were kindly provided by Mash Premiere Pharmaceutical Company, Badr City, Cairo, Egypt. Other materials, were utilized through this work are in the analytical grade. Acetic acid, acetone, chloroform, carboxymethylcellulose sodium, dichloromethane, ethyl acetate, ethanol, hexane, methanol, polyethylene glycol 6000 orthophosphoric acid, sulfuric acid, Tween-80, sodium dodecyl sulfate, and sodium hydroxide, were purchased from El-Nasr Chemicals Company, Cairo, Egypt. Acetonitrile, beta-cyclodextrin, dimethyl sulphoxide, Hexadecyl trimethyl ammonium bromide, and Triton X-100 were purchased from Sigma Aldrich, St. Louis, USA. Sofolanork plus® commercial tablets (contain 90 mg of LDS and 400 mg of SOF per tablet), were kindly provided by Mash Premiere Pharmaceutical Company, Badr City, Cairo, Egypt. Swiss-Albino male rats with an average weight of 200–260 g were obtained from Center of Experimental Animal at College of Medicine, Assiut University, Assiut, Egypt. The protocol was conducted in accordance with the ethical standards approved by the Institutional Animal Ethics Committee guidelines for animal care and use, Minia University, Egypt. LDS was orally administered to rats once daily in a dose of 9 mg kg^−1^.^6^

### 2.3. Quality control (QC) samples and standard solutions

A standard stock solution of both LDS and SOF were prepared by dissolving the weighed authentic material in methanol to prepare standard solutions of each drug at a concentration level of 100 μg mL^−1^. The obtained solutions were appropriately diluted with methanol to prepare LDS and SOF working solutions. Calibration standards of LDS in faecal and plasma samples were prepared by spiking the working solution of LDS into drug-free faeces samples and drug-free plasma samples, respectively at the following concentrations: 30, 50, 80, 100, 120 and 150 ng mL^−1^. The quality control (QC) samples of LDS working solutions were prepare at high, medium and low concentration levels (120, 80, 40 ng mL^−1^).^17^ All containers of LDS solutions (quality control samples, calibration standards, working solutions, and stock solutions) were wrapped immediately by aluminum foil and stored in the refrigerator at −20 °C. All of them were thawed before the analysis time at room temperature.

### 2.4. General analytical procedure

Known volumes of LDS solution in the concentration range of 0.05 to 1.5 μg mL^−1^ were poured to volumetric flasks (10 mL). The flasks were placed in a dark black iron box part of UV-transilluminator and exposed to the direct UV irradiation for 15 min. Then volumetric flasks were totalled up to the line marker with ethanolic H_3_PO_4_ (7% v/v) solution. Eventually; the fluorescence intensity (FI) of the resulted solution was recorded at an emission wavelength of 375 nm (321 nm excitation). Blank measuring was achieved by carrying out the same analytical procedures with omitting the cited drug.

### 2.5. Samples preparation

#### 2.5.1. Pharmaceutical tablets

Seven Sofolanork plus® tablets were precisely weighed and crushed in a porcelain mortar. A quantity of the powdered tablets (equivalent to 10.0 mg LDS) was transferred into a volumetric flask (100 mL). The powder was sonicated for 15 min with 30 mL methanol, then the volume of the flask was completed up to the marker with the same solvent to achieve LDS in a concentration level of 100 μg mL^−1^. The resulted solution was filtered and the initial section of the filtrate was rejected. The obtained solution was subjected to adequate dilution with methanol to make the final concentration of LDS in the calibration range. The general analytical procedures were carried out as mentioned in Section 2.4 using five replicate from the extraction of tablets sample.

#### 2.5.2. Pharmacokinetic study and plasma samples

After the first oral administration of LDS for rats group, the blood samples (1.0 mL) were drawn from tail vein of the rat and transferred to 2 mL heparinized tube at time intervals of 0.25, 0.5, 1.0, 1.5, 2.0, 3.0, 4.0, 6.0, 8.0, 10.0, 12.0 and 24.0 h. The samples were immediately centrifuged for 5 min at 10 000 g, after that, the plasma part was transferred into a 2 mL centrifuge tube and deproteinized by 0.5 mL of acetonitrile then vortexed for about one min and then centrifuged at 10 000 g for five min. Thereafter, the general analytical procedures were applied to the supernatant part of the plasma samples as mentioned in Section 2.4.

#### 2.5.3. Faecal samples preparation

One-day collection of rats faeces was homogenized and vortexed for 5 min with an appropriate amount of distilled water in 50 mL Falcon™ tube. The sample volume was totalled up to 30 mL by distilled water. Five milliliters of the homogenized faeces sample was transferred into 15 mL Falcon™ tube and vortexed with two milliliters of acetonitrile for 5 min then centrifuged at 4000 g for 20 min. Thereafter, the general analytical procedures were applied to the supernatant part as mentioned in Section 2.4.

### 2.6. Procedures for content uniformity test

Content uniformity test for LDS in the pharmaceutical tablets dosage form was applied according to USP guidelines (Chapter 905).^18^ The testing for uniformity of dosage units in this work was performed by the individual analysis of ten Sofolanork plus® tablets utilizing the analytical procedures described under the section of pharmaceutical tablets preparation.

## 3. Results and discussion

### 3.1. Photochemical reaction and strategy of assay

**Fig. 2:**
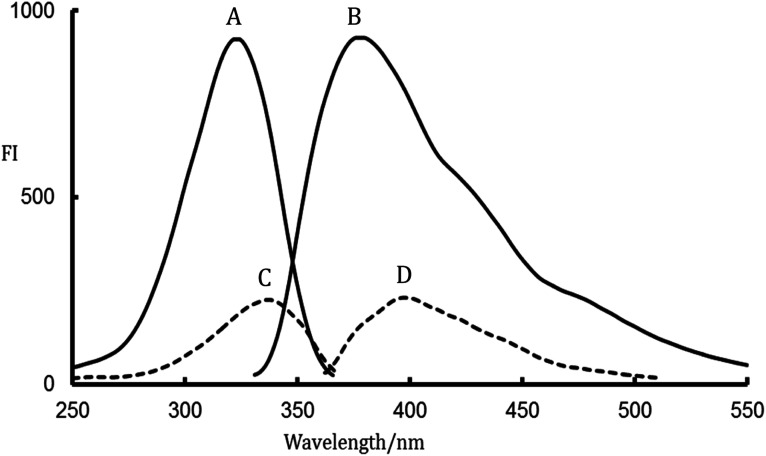
Fluorescence spectra; A, B are excitation and emission spectra for irradiated-LDS, respectively and C, D are excitation and emission spectra for intact LDS, respectively.

The photodegradation of different compounds bearing fluorine atoms on their aromatic skeleton was studied in previously reported works.^19–21^ It was known well that the defluorination of aromatic systems leads to a great boost in the fluorescence of the resulting compounds. Accordingly, an improvement in sensitivity of the analytical methods was achieved.^22,23^ LDS contains two fluorine atoms in its fluorenyl moiety which suppresses the fluorescence properties of the studied drug, Fig. 1. LDS undergo a photolytic degradation when exposed to strong direct UV irradiation leads to defluorination and rigidification of the studied drug *via* conversion fluorene ring to fluorenone ring.^9^ This process improves both the sensitivity (400% enhancement of LDS FI) and specificity (only LDS liable for UV-degradation in samples) for determination of the cited drug with wide space of applications. The photodegradation of LDS can be easily observed by the naked eye after exposing the methanolic solution of LDS with concentrations higher than 10 μg mL^−1^ to direct sunlight irradiation or UV-transilluminator combined with a colour change from colourless to the yellow colour. The defluorination process not only boosts the fluorescence intensity of aromatic systems but also change the peak position.^22,23^ The fluorescence spectra for irradiated-LDS [the major photolytic degradation product (PDP)] and intact-LDS are shown in Fig. 2. It can be seen from the emission and excitation spectra that a great enhancement in the fluorescence intensity (FI) for the irradiated LDS with a blue shift of the emission band more than non-irradiated LDS. Recently, the photolysis degradation of LDS was studied in detail.^9,13^ The major PDP was separated (impurity F, formed *via* the defluorination pathway^9^), identified and characterized by ^1^HNMR, IR, MS spectroscopy, and LC-MS/MS.^9,13^ The defluorination mechanism was illustrated in Fig. 1.^9^

### 3.2. Optimization of experimental factors

Various experimental conditions were superintended and optimized to obtain the greatest fluorescence intensity values for PDP.

#### 3.2.1. Influence of irradiation time

**Fig. 3:**
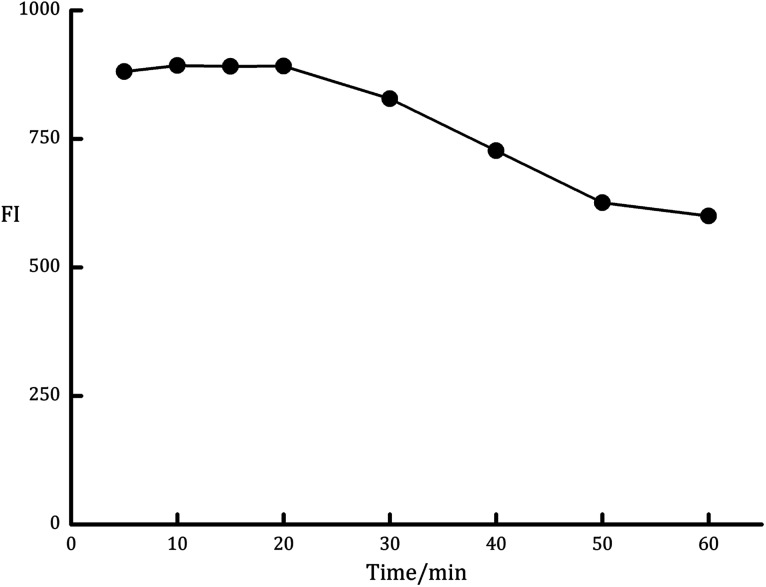
Effect of irradiation time on the fluorescence intensity of the PDP of ledipasvir (100 ng mL^−1^).

The degree of improvement in the fluorescence intensity (FI) of LDS was highly depending on the time of UV irradiation. The effect of this variable was examined over the time intervals of 5 to 60 min (Fig. 3). It was observed that the optimal irradiation time that gives the maximum FI was 10 to 20 min. The longer time is not recommended as the fluorescence intensity was gradually decreased by increasing the exposure time. Therefore, 15 min irradiation period was chosen for the analysis of LDS in the presented study.

#### 3.2.2. Organic solvents effect

**Fig. 4:**
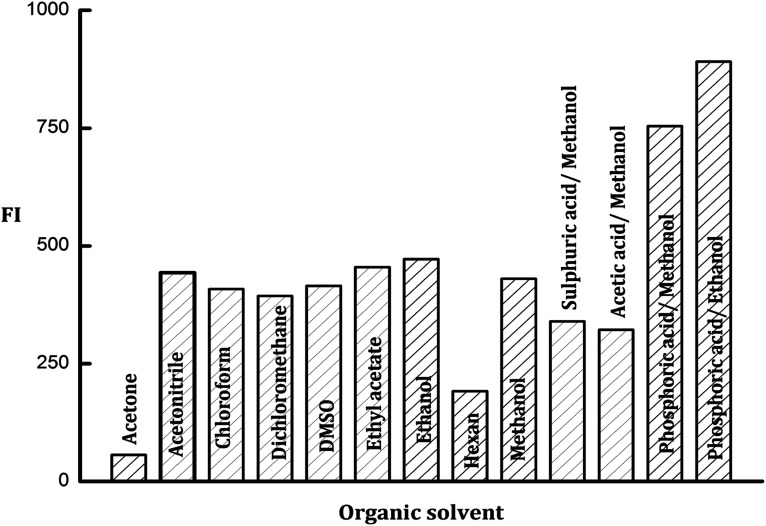
Effect of different types of organic solvents and acids on the fluorescence intensity of the PDP of ledipasvir (100 ng mL^−1^).

Many organic solvents were investigated for the enhancement of the fluorescence emission intensity of the formed PDP. The tried solvents were acetone, acetonitrile, chloroform, dimethyl-sulphoxide, dichloromethane, ethyl acetate, ethanol, hexane, and methanol. It was observed that the fluorescence intensity of the formed PDP gave the lowest results with acetone and hexane while the maximum FI values were achieved with methanol and ethanol. The results of other organic solvents were slightly lower than ethanol as shown in Fig. 4.

#### 3.2.3. Influence of organized media

**Fig. 5:**
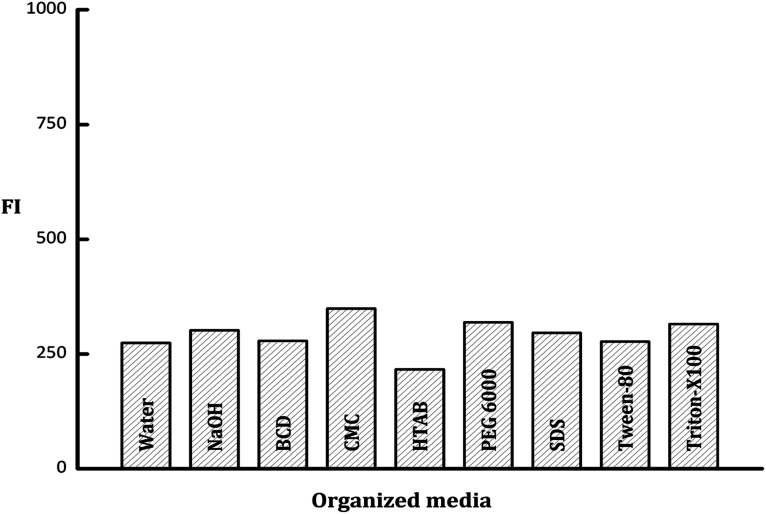
Effect of various types of organized media, water and NaOH on the fluorescence intensity of the PDP of ledipasvir (100 ng mL^−1^).

In a trial to enhance the fluorescence intensity of PDP, different micellar media were investigated including; sodium dodecyl sulfate (SDS, 1 × 10^−2^ mol L^−1^), polyethylene glycol 6000 (PEG 6000, 1% w/v), beta-cyclodextrin (β-CD, 1% w/v), Tween-80, (1% v/v), hexadecyl trimethyl ammonium bromide (HTAB, 1 × 10^−2^ mol L^−1^), carboxymethylcellulose sodium (CMC Na, 1% w/v), and Triton X-100 (1% v/v). It was founded that the emission intensity value of PDP did not impact by the studied organized media with a slight enhancement observed with CMC Na and Triton-X100 (Fig. 5). Consequently, neither of these surfactants was incorporated in the general analytical procedure of the current work.

#### 3.2.4. Influence of acids

Various acids in alcoholic media were tried included; methanolic acetic acid, methanolic sulfuric acid, methanolic orthophosphoric acid and ethanolic orthophosphoric acid solutions (all of them were prepared in one molar concentration). The obtained results indicated that there is no impact on the emission intensity of the formed PDP neither from acetic acid nor sulfuric acid solutions. On the other hand, the great enhancement in FI of PDP was obtained with orthophosphoric acid in methanolic or ethanolic solutions (Fig. 4). Thus ethanolic orthophosphoric acid was chosen for subsequent tests.

### 3.3. Method validation

The proposed method was validated according to ICH guidelines with respect to linearity, sensitivity, selectivity, robustness, precision, and accuracy.^24^ Moreover, the developed bio-analytical procedure was validated according to FDA guidelines.^25^

#### 3.3.1. Range and linearity

Series of eight standard solutions of LDS were analyzed to assess the linearity for the proposed method. The calibration curve was constructed by plotting the resulted FI values of the irradiated solution against final concentrations of LDS in ng mL^−1^. It was found that the calibrated linearity was ranged from 5 to 150 ng mL^−1^. Also, the statistical parameters for the proposed method were statistically come by analyzing the resulted linear regression as shown in Table 1. In the case of biological samples (bio-analytical procedure) the calibration curves for both plasma and faecal samples were originated by six various concentration levels in the range of 30–150 ng mL^−1^. The resulted regression equations were *y* = 7.749*x* + 124.62 and *y* = 8.047*x* + 87.80 for faecal and plasma samples, respectively with correlation coefficients (*r*^2^) of 0.9996 and 0.9990 for faecal and plasma samples, respectively.

**Table:** Analytical performance data for the proposed method

Parameter	Value
Linear range (ng mL^−1^)	5–150
Slope	7.91
SD of slope (*S*_b_)	0.028
Intercept	115.51
SD of intercept (*S*_a_)	2.16
Determination coefficient (*r*^2^)	0.9998
Correlation coefficient (*r*)	0.9999
Number of determinations	8
SD of the residuals (*S*_*y*/*x*_)	4
Limit of quantitation (ng mL^−1^)	2.7
Limit of detection (ng mL^−1^)	0.9

#### 3.3.2. Sensitivity parameters

According to ICH rules, LOQ and LOD values were determined by adopting equations: LOQ = 10 *S*/*b* and LOD = 3.3 *S*/*b*, where; *S* is the standard deviation of the intercept and *b* is the slope of the calibration curve. The resulted LOQ and LOD values were 2.7 and 0.9 ng mL^−1^, respectively. These achieved values indicate to superior sensitivity of the proposed method with its ability for the determination of LDS in plasma and faecal samples.

#### 3.3.3. Precision and accuracy

The accuracy was assigned by analyzing five concentrations (20, 40, 80, 100 and 120 ng mL^−1^) of standard LDS solutions by the proposed method. Inter-day precision was evaluated by analyzing three concentration levels of standard LDS solution (25, 75 and 125 ng mL^−1^) for three consecutive days. Whilst, intra-day precision was evaluated by analyzing three concentration levels of the standard LDS solution (25, 75 and 125 μg mL^−1^) within the same day. Both accuracy and precision analysis were performed in three replicates for each concentration levels. As offered in Table 2, the adequate accuracy level of the proposed method was provided by closeness the resulted percent recovery values to the true value (one hundred percent). Also, the obtained data from Table 2, pointed out to the adequate precision of the proposed method at intra-day and inter-day precision levels which proved by the low values of the resulted relative standard deviation.

Validation parameters for the analysis of LDS in pure form by the proposed method

**Table:** 

Parameter	ng mL^−1^	% Recovery ± SD
Accuracy^a^	20.0	99.54 ± 1.26
	40.0	99.49 ± 1.42
	80.0	100.25 ± 1.58
	100.0	98.36 ± 0.60
	120.0	100.37 ± 1.85

a Mean of three determinations, SD: standard deviation, RSD: relative standard deviation.

b Mean of three replicates of 1 μg mL^−1^.

**Table:** 

Parameter	μg mL^−1^	% Recovery ± RSD
Intra-day precision^a^	25.0	98.84 ± 1.84
	75.0	100.36 ± 0.61
	125.0	101.51 ± 0.30
Inter-day precision^a^	25.0	99.35 ± 1.02
	75.0	101.83 ± 0.91
	125.0	101.24 ± 0.76

**Table:** 

Parameter	Irradiation time	% Recovery ± SD
Robustness^b^	15 mn	101.06 ± 0.63
	10 mn	98.74 ± 0.97
	20 mn	98.28 ± 0.99

#### 3.3.4. Robustness

The stability of FI values was used as a monitor to assign the robustness of the proposed method across the deliberated minor changes in the optimized experimental factors. Fortunately, the general analytical procedure of the proposed method is very simple and the changes in the time of irradiation parameter (15 ± 5 min) did not have any considerable effect on FI values which prove that the method is robust (Table 2).

#### 3.3.5. Specificity and selectivity

The selectivity was assigned by studying the effect of pharmaceutical excipients on the recoveries of the proposed method. The study included magnesium stearate, starch, glucose, lactose, sorbitol, and talc. The presented results in Table 3 indicate that there is no interference from these excipients on the proposed analytical procedures as confirmed by the resulted percent recoveries. Furthermore, among other co-formulated or co-administrated anti-HCV drugs, tablet excipients and biological matrices only LDS was liable for UV-degradation with distinctive enhancement in the fluorescence properties. In addition, the specificity of the proposed method was tested by analysis of synthetic mixtures containing LDS and SOF. The mean of recoveries and RSD for LDS determination in the synthetic mixtures were 100.09 and 1.25, respectively (Table 4), that means the absence of interference from SOF at high or low concentration levels of both drugs.

**Table:** Analysis of LDS in the presence of some common excipients using the proposed method

Excipients	Amount added (μg mL^−1^)	LDS con. (ng mL^−1^)	% Recovery ± SD^a^
Starch	50	100	100.22 ± 1.32
Mg stearate	10	100	99.8 ± 1.67
Lactose	10	100	98.74 ± 0.97
Glucose	10	100	101.48 ± 1.59
Talc	5	100	101.69 ± 0.63
Sorbitol	10	100	98.53 ± 1.26

a Mean of three replicate measurement, SD: standard deviation.

**Table:** Assessment the specificity for the proposed method by analysis of LDS in binary mixtures with SOF

LDS conc	SOF conc	LDS conc	(Recovery ± SD^a^) for LDS
25.0	150.0	24.753	99.01 ± 1.63
75.0	100.0	74.726	99.63 ± 1.29
100.0	75.0	100.639	100.64 ± 0.97
150.0	25.0	151.623	101.08 ± 1.11
Mean			100.09
RSD			1.25

a Mean of three replicate measurement, drugs conc (ng mL^−1^), SD: standard deviation, RSD: relative standard deviation.

### 3.4. Analysis of pharmaceutical tablets

The proposed method was applied for the determination of LDS in its pharmaceutical dosage form (Sofolanork plus® tablets). The percentage (recovery ± SD) was 100.50 ± 1.01 for the proposed method. Student's *t*-test and variance ratio *F* test were statistically estimated for comparing the resulted recovery values from both the proposed and the reported spectrofluorimetric methods.^13^ As offered in Table 5, we can found that the calculated values of the proposed method were lower than the theoretical values, and no considerable difference between the recovery values achieved by both methods. This results evidencing similar precision and accuracy for LDS analysis by the mentioned spectrofluorimetric methods.

**Table:** Estimation of LDS in pharmaceutical tablets by proposed and reported spectrofluorimetric methods

Dosage form	% Recovery^a^ ± SD		*t*-Value^b^	*F*-value^b^
	Proposed	Reported		
Sofolanork plus® tablets	100.50 ± 1.01	98.88 ± 1.60	1.91	2.5

a Average of five determinations.

b Tabulated value at 95% confidence limit; *F* = 6.338 and *t* = 2.306.

### 3.5. Content uniformity test

The content uniformity test (CU) can be applied to check uniformity of dosage units when the drug-substance ratio <25% or when the drug-substance dose <25 mg.^18,26^ The average ratio of LDS in Sofolanork plus® tablets was 6.92%. Therefore, the uniformity of dosage units for LDS can be monitored by the CU test as described in official USP guidelines.^18^ The procedures for the CU test could suffer from the prolong time of operation when utilizing complicated analytical techniques.^27^ However, the proposed method has a rapid, simple and specific procedure for measuring the FI of LDS in tablets over than all the reported HPLC^7^ and UV-spectrophotometric^11^ methods for monitoring this test. Hence, the proposed method is extremely proper for this purpose. According to official USP guidelines, the CU test can be estimated by calculation the acceptance value (AV) for tablets.^18^ AV was estimated by adopting the equation:^18^ AV = |*M* − *X̄*| + *KS*, where; *M* is a reference value, *X̄* is the mean of the individual content, *K* is the acceptability constant, and *S* is the standard deviation of samples. The requirement for accepting the CU of tablets dosage units is: AV of ten tablets is ≤L1 (the maximum allowed acceptance value). It was found that the calculated AV for Sofolanork plus® by the proposed method was lower than the L1 value. The obtained results in Table 6 indicated the acceptable content uniformity for LDS in its commercial tablets.

**Table:** Results of the content uniformity test for Sofolanork® plus commercial LDS tablets^a^

Tablet number	% Recovery
1	99.16
2	101.06
3	101.69
4	99.80
5	100.43
6	97.90
7	98.53
8	97.90
9	99.16
10	104.85
Mean (*X̄*)	99.80
*S*	2.11
AV*	8.63
L1*	15

a L1: maximum allowed acceptance value, AV: acceptance value.

### 3.6. Analysis of biological samples

**Fig. 6:**
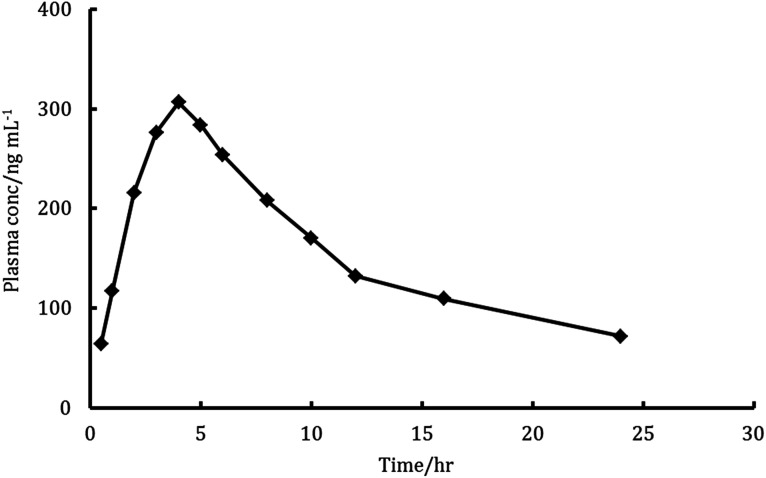
Plasma concentration–time profiles curve in rats after a single oral dose of 9 mg kg^−1^ ledipasvir.

The major elimination pathway for LDS was the biliary route^5^ and the major excretion route for the radioactive-LDS was *via* the faecal excretion as unchanged parent drug, with about 86% of the orally taken dose of LSD has been recovered. Meanwhile, about 1% of the dose was excreted in urine.^5^ These data and the excellent sensitivity and specificity features of the proposed method encouraged us to investigate the amounts of unchanged parent drug in rats plasma and faeces utilizing the proposed method. The mean recovery for determination of LDS in faeces samples was 90.4 ± 4.1 and the mean plasma profiles of concentration–time was presented in Fig. 6. The *C*_max_ for LDS (maximum plasma concentration) found to be 306.7 ng mL^−1^ and achieved after 3.8 hours. The area under the curve (AUC_0–*t*_) was found to be 3682 ng h mL^−1^. The AUC_0–∞_ was found to be 5911 ng h mL^−1^. Also, the elimination half lifetime (*t*_1/2_) was found to be 21.6 hour. The achieved results were similar to those achieved with the reported UPLC-MS/MS chromatographic method for the determination of LDS in rat plasma.^6^ Some of the achieved results were quite high, this may be related to the omission of extraction steps in the proposed method. In the previously reported chromatographic method, the presence of multistep extraction in the analytical procedures was combined with the high risk of LDS losses. Thus, the proposed method could provide a superior alternative for efficient quantification of LDS in biological samples.

## 4. Conclusion

This research offers an innovative spectrofluorimetric method for the estimation of ledipasvir in various types of samples as well as monitoring the content uniformity of dosage units. This method did not require any critical chemical reagents, instead, the physical irradiation has been utilized to improve the sensitivity of the proposed method. In addition, the presented work has given a number of advantages involving specificity, sensitivity, short time of analysis, uncomplicated analytical steps, lowering the reagents and instrumental costs. Therefore, this fluorometric method can be considered as a great benefit in clinical, laboratories and industrial scales.

## Conflicts of interest

There are no conflicts to declare.

## Supplementary Material

## References

[cit1] Lee M.-H., Yang H.-I., Yuan Y., L'Italien G., Chen C.-J. (2014). World J. Gastroenterol..

[cit2] Abdel-Lateef M. A., Omar M. A., Ali R., Derayea S. M. (2018). Microchem. J..

[cit3] Sharma N. K. and Sherker A. H., in Chronic Viral Hepatitis, Springer, 2009, pp. 33–70

[cit4] Afdhal N., Reddy K. R., Nelson D. R., Lawitz E., Gordon S. C., Schiff E., Nahass R., Ghalib R., Gitlin N., Herring R., Lalezari J., Younes Z. H., Pockros P. J., Di Bisceglie A. M., Arora S., Subramanian G. M., Zhu Y., Dvory-Sobol H., Yang J. C., Pang P. S., Symonds W. T., McHutchison J. G., Muir A. J., Sulkowski M., Kwo P. (2014). N. Engl. J. Med..

[cit5] German P., Mathias A., Brainard D., Kearney B. P. (2016). Clin. Pharmacokinet..

[cit6] Pan C., Chen Y., Chen W., Zhou G., Jin L., Zheng Y., Lin W., Pan Z. (2016). J. Chromatogr. B: Anal. Technol. Biomed. Life Sci..

[cit7] EL-Shorbagy H. I., Elsebaei F., Hammad S. F., El-Brashy A. M. (2019). Microchem. J..

[cit8] Abdallah O. M., Abdel-Megied A. M., Gouda A. S. (2017). J. Pharm. Biomed. Anal..

[cit9] Siva Kumar R., Sravan Kumar K., Kondareddy L., Yogeshwara K., Manish G., Jeenet J., Nitesh K. (2018). J. Chromatogr. Sci..

[cit10] Mansour F. R. (2018). Spectrochim. Acta, Part A.

[cit11] EL-Shorbagy H. I., Elsebaei F., Hammad S. F., Elbrashy A. M. (2018). Spectrochim. Acta, Part A.

[cit12] Salama F. M., Attia K. A., Abouserie A. A., El-Olemy A., Abolmagd E. (2018). Spectrochim. Acta, Part A.

[cit13] Ali R., Marzouk A. A., Abdelhameid R. A., Omar M. A. (2018). Spectrochim. Acta, Part A.

[cit14] Abo-Zeid M. N., Atia N. N., El-Gizawy S. M., El-Shaboury S. R. (2018). J. Pharm. Biomed. Anal..

[cit15] Omar M. A., Derayea S. M., Abdel-Lateef M. A., El Hamd M. (2018). Spectrochim. Acta, Part A.

[cit16] Omar M. A., Abdel-Lateef M. A., Ali R., Derayea S. M. (2018). Luminescence.

[cit17] Abdel-Lateef M. A., Omar M. A., Ali R., Derayea S. M. (2019). Spectrochim. Acta, Part A.

[cit18] United States , Pharmacopoeia 30th and The National Formulary 25th, Electronic Version Rockville, Maryland, USA, 2007

[cit19] Lam M. W., Young C. J., Mabury S. A. (2005). Environ. Sci. Technol..

[cit20] Cydzik I., Albert-Garcia J., Calatayud J. M. (2007). J. Fluoresc..

[cit21] Sturini M., Speltini A., Maraschi F., Pretali L., Profumo A., Fasani E., Albini A., Migliavacca R., Nucleo E. (2012). Water Res..

[cit22] Bronskill P. M., Wong J. (1988). Biochem. J..

[cit23] Becker R. S. and Becker R. S., Theory and interpretation of fluorescence and phosphorescence, Wiley Interscience, New York, 1969

[cit24] ICH Harmonised Tripartite Guideline Q2 (R1), Validation of analytical procedures. Text and methodology, current step 4 versions, parent Guideline on methodology dated November 1996, Incorporated in November 2005

[cit25] FDA, Guidance for industry: bioanalytical method validation. US Department of Health and Human Services, Food and Drug Administration, Center for Drug Evaluation and Research, Center for Veterinary Medicine 2001, http://www.fda.gov/downloads/Drugs/Guidances/ucm070107.pdf

[cit26] Abdel-Lateef M. A., Omar M. A., Ali R., Derayea S. M. (2019). Vib. Spectrosc..

[cit27] Derayea S. M., Omar M. A., Abdel-Lateef M. A.-K., Hassan A. I. (2016). Open Chem..

